# Supplementary material for: Structure of a mitochondrial ribosome with fragmented rRNA in complex with membrane-targeting elements
Source: Nat Commun. 2022 Oct 17;13:6132. doi: 10.1038/s41467-022-33582-5 (PMC9576764; doi:10.1038/s41467-022-33582-5)
Supplement: Supplementary file 1 — Supplementary Information [file 41467_2022_33582_MOESM1_ESM.pdf]

Structure of a mitochondrial ribosome with fragmented rRNA in complex with membrane-targeting elements

Victor Tobiasson[1], Ieva Berzina[2], Alexey Amunts[1,*]

[1]Science for Life Laboratory, Department of Biochemistry and Biophysics, Stockholm University, 17165 Solna, Sweden
[2]Department of Medical Biochemistry and Biophysics, Karolinska Institute, 17177 Stockholm, Sweden

[*] Correspondence to: amunts@scilifelab.se

SUPPLEMENTARY INFORMATION

<u>Content of SI:</u>

Supplementary Figures 1-8

Supplementary Tables 1-2

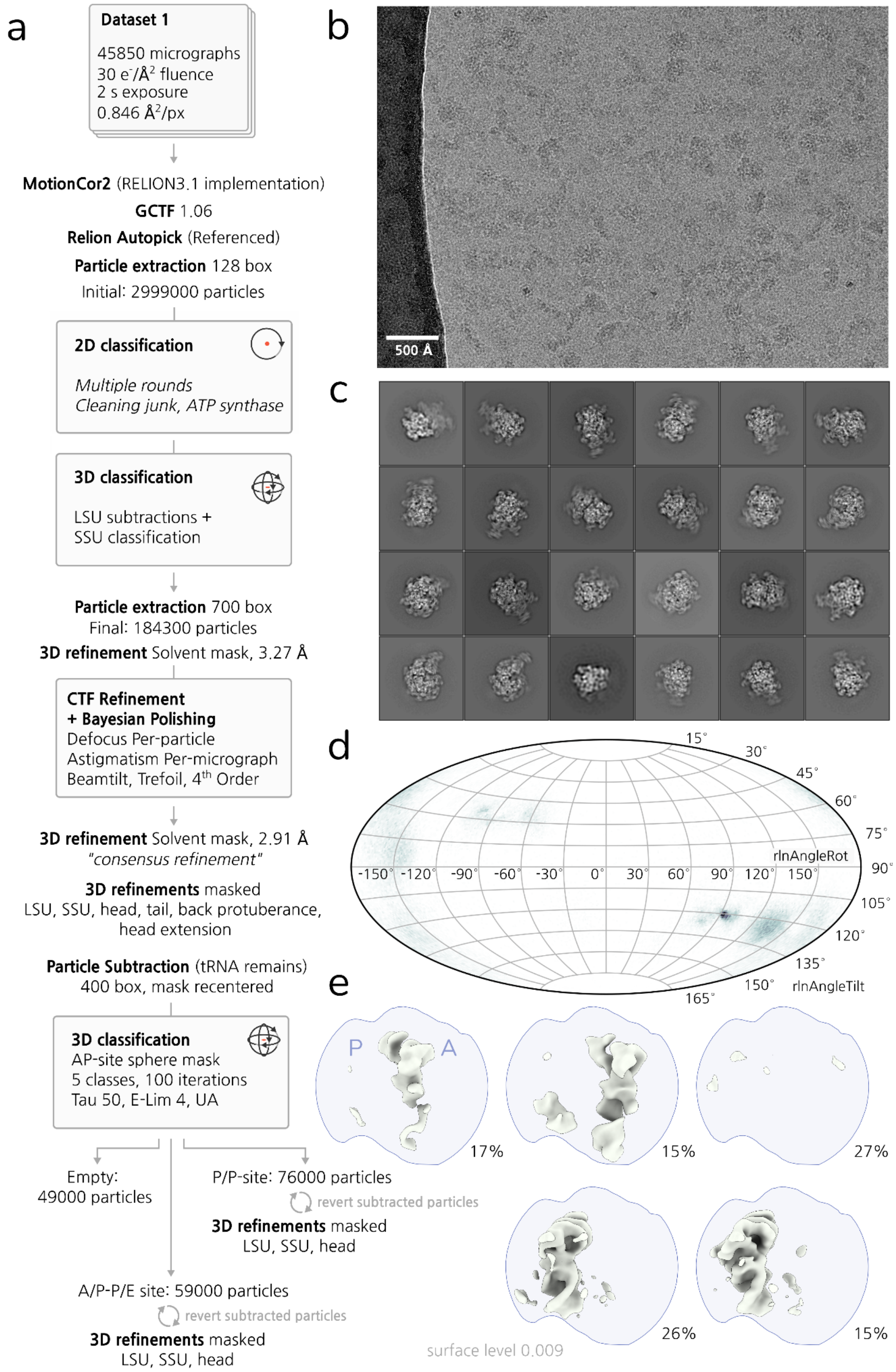

Supplementary Fig. 1: Cryo-EM processing workflow.
a, Flowchart of data processing. **b,** Representative micrograph. **c,** Final 2D classes from 184300 particles. **d,** Histogram of angular distribution calculated for consensus refinement, modified azimuthal projection. **e,** Final classification of tRNA subvolumes including classification mask outline in blue.

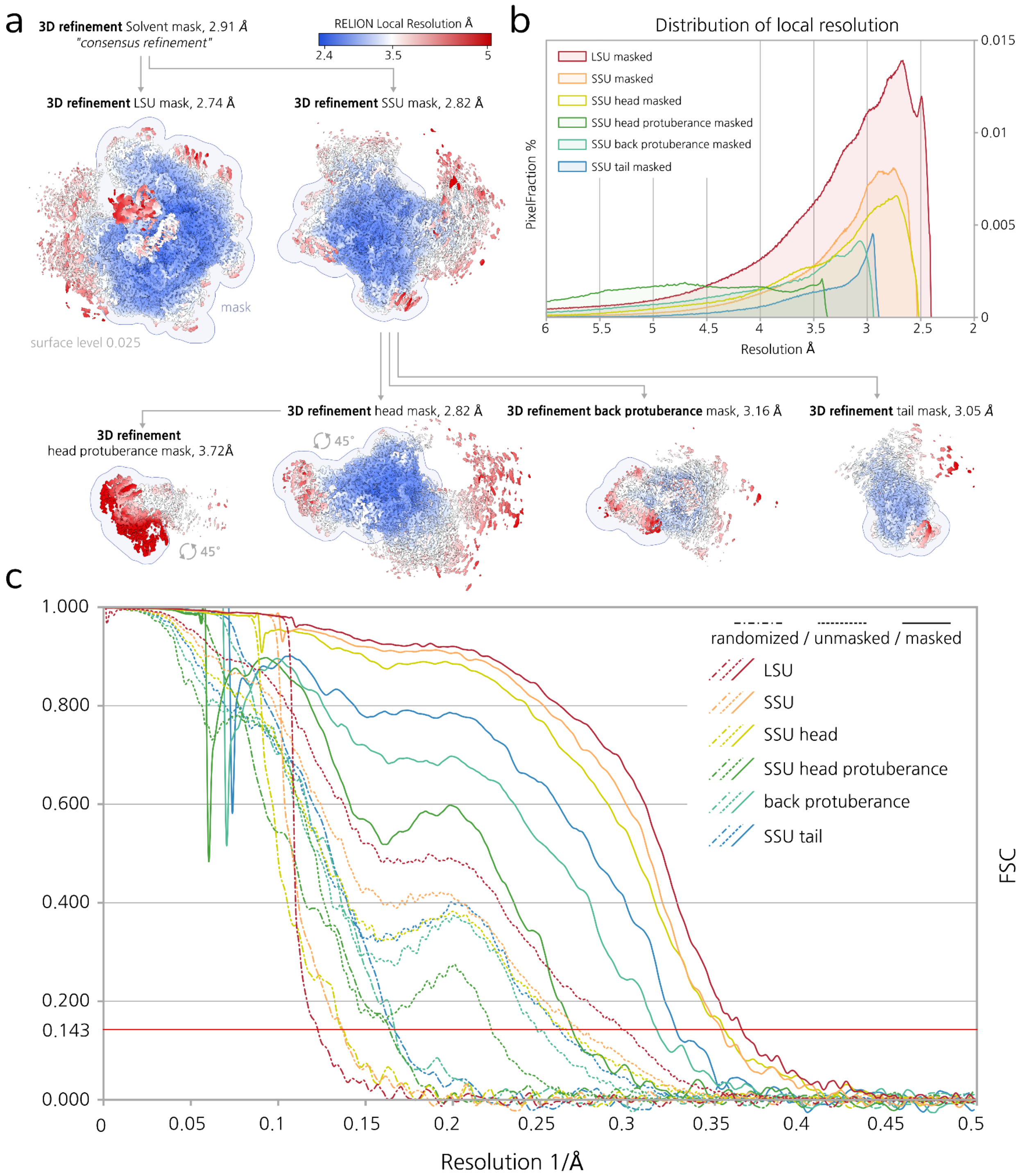

Supplementary Fig. 2: Cryo-EM map resolutions.

a, Refinement scheme for masked refinements from consensus particle subset. Local resolution filtered maps shown, surface colored by local resolution values. Masks used for refinement and local resolution calculation shown in blue. Arrow sources of initial angles for refinement. **b,** Histogram of local resolution voxel values, counts normalised by number of voxels within the provided mask. **c,** FSC curves for masked refinements shown. Dotted lines indicate unmasked FSCs and dashed lines indicate phase randomised FSCs.

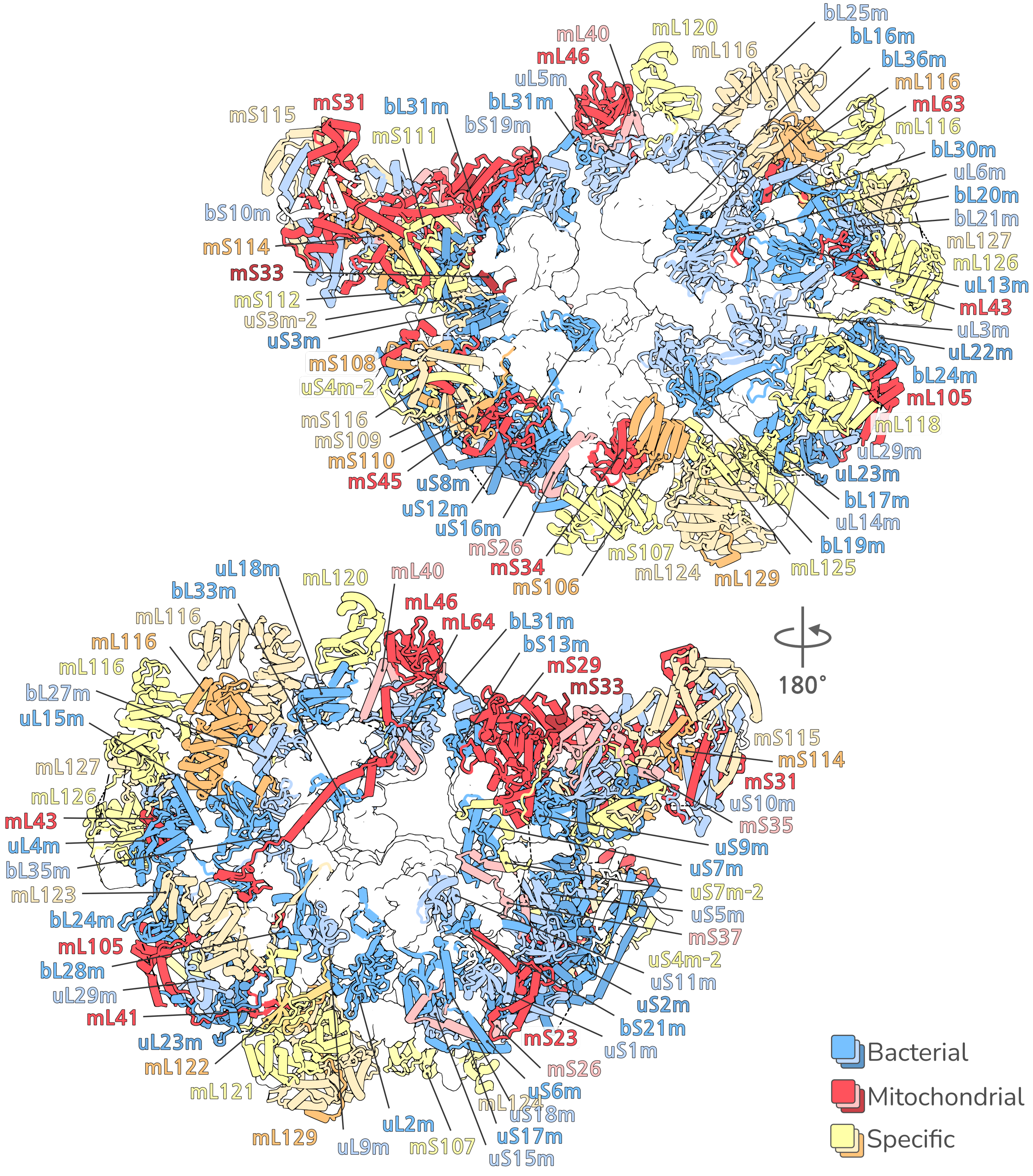

Supplementary Fig. 3: *P. Magna* protein conservation.
View of the monosome with proteins colored by conservation. Three shades of blue indicate proteins with known bacterial homologs. Proteins conserved across mitochondrial ribosomes colored in shares of red. Lineage-specific proteins colored in shades of yellow.

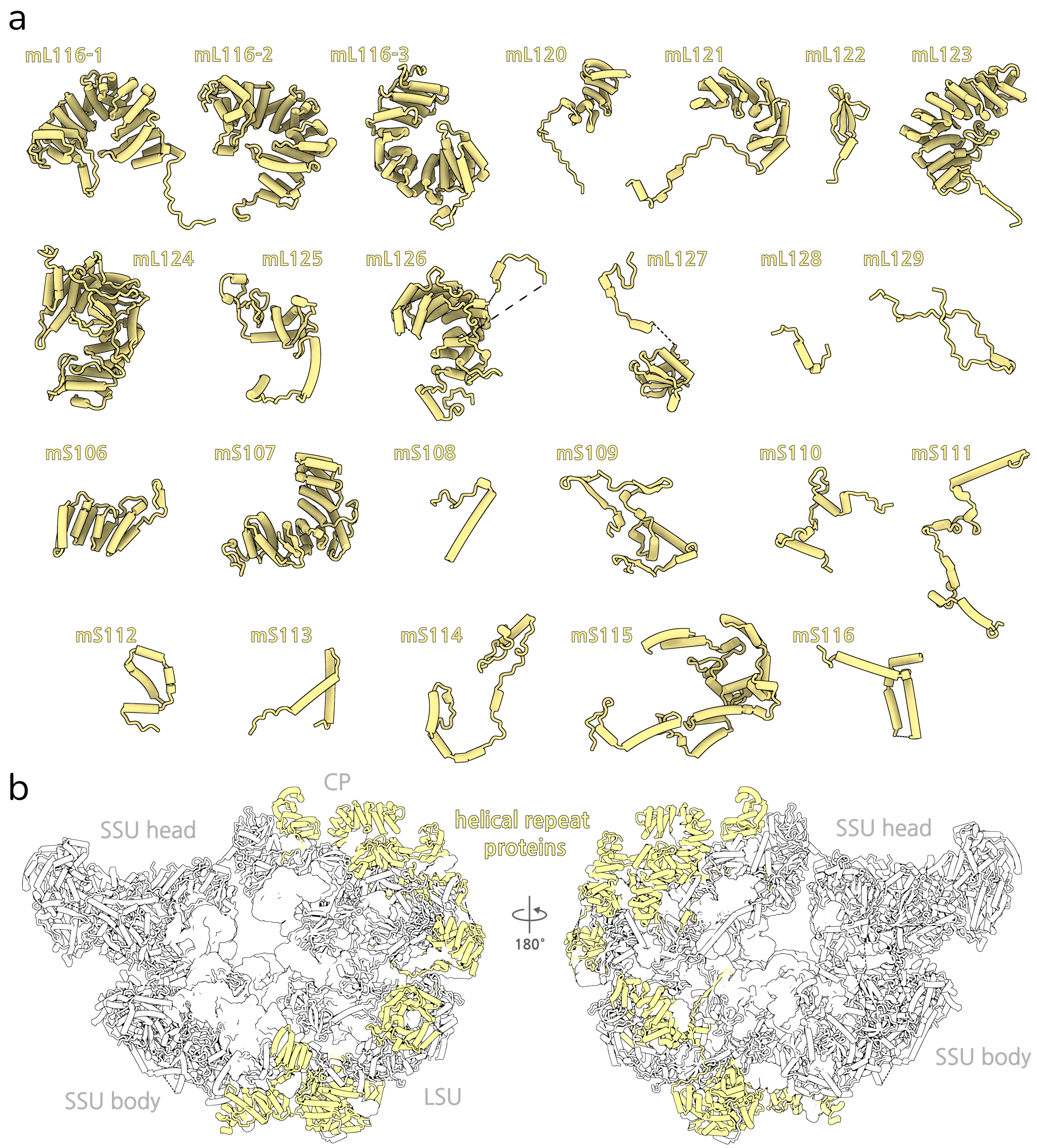

Supplementary Fig. 4: Lineage specific proteins and HEATs in *P. magna*
a, Proteins present in only either the current *P. magna* structure or the structure from *C. reinhardtii.* **b,** Distribution of HEAT proteins on the P. magna mitochondrial ribosome.

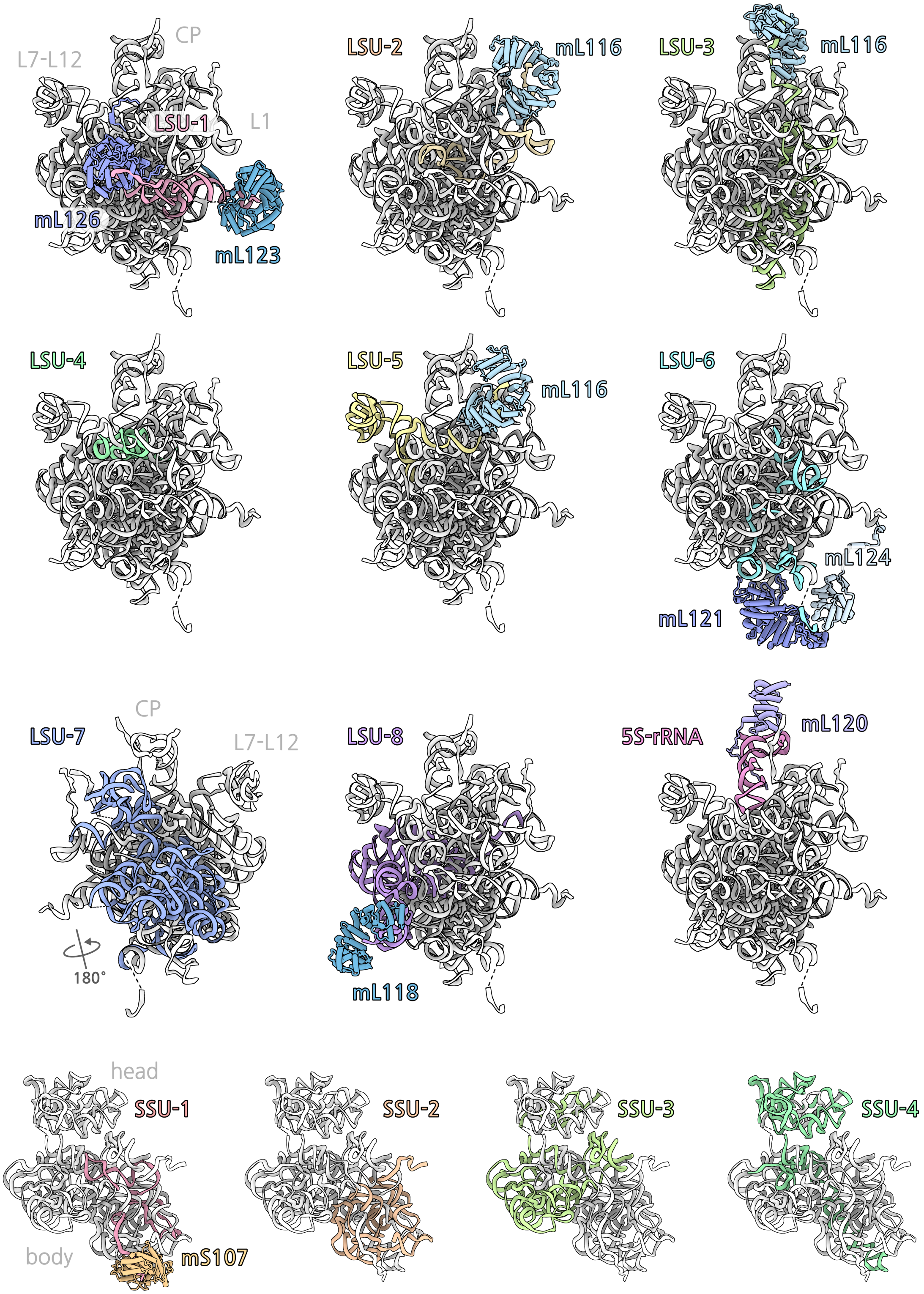

Supplementary Fig. 5: Relative position of rRNA fragments and bound HEAT proteins. Each rRNA fragment is coloured individually with its associated HEAT protein, remaining rRNA in white.

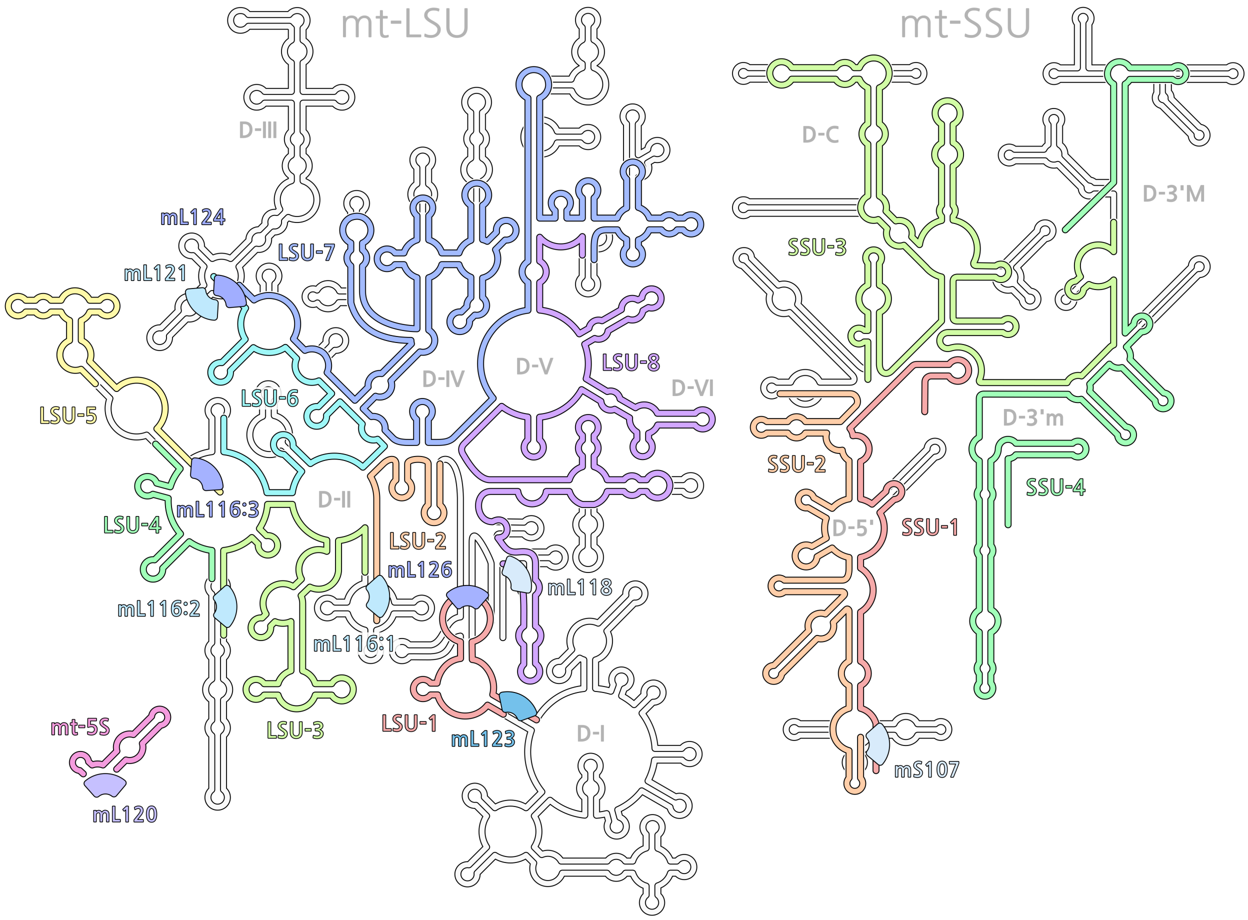

Supplementary Fig. 6: Schematic map of the binding of HEAT proteins to rRNA.
The rRNA of LSU (left) and SSU (right) fragments colored sequentially, superimposed onto *E. coli* rRNA in white. Binding locations of eleven HEAT-repeat proteins indicated.t

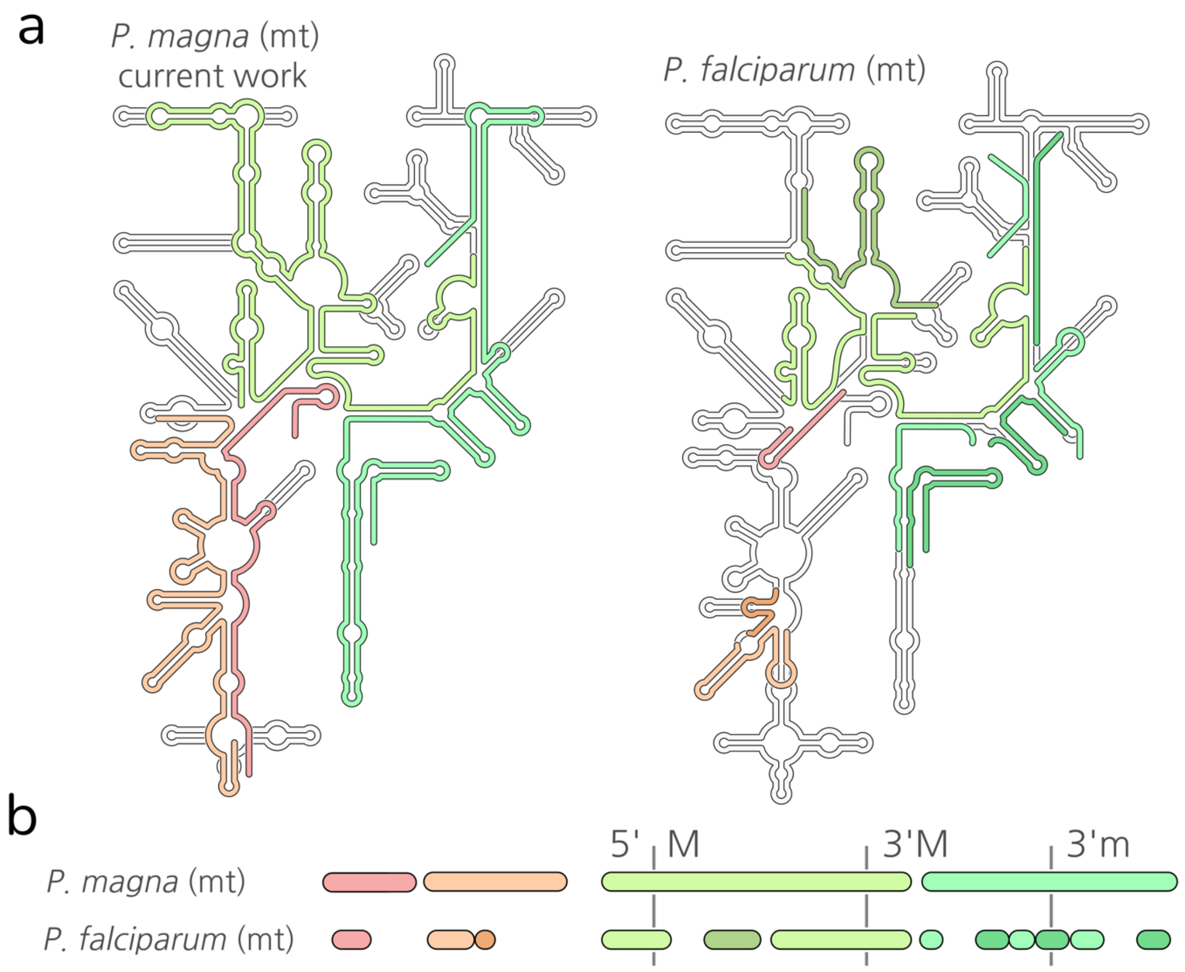

Supplementary Fig. 7: SSU rRNA fragmentation patterns in *P. magna* and *P. falciparum*. **a,** Fragmentation patterns of the *P. magna* and *P. falciparum* mitochondrial SSUs highlighting the subfragmentation patterns between mitochondrial ribosomes. *T. brucei* and *E. gracilis* omitted as they are not fragmented. Fragments spanning equivalent regions of the rRNA secondary structures coloured by hue, individual fragments highlighted by colour brightness. E.coli reference diagram as a white background. **b**, Linear representation of fragment span. Colored according to **a,** Lengths semi-conserved.

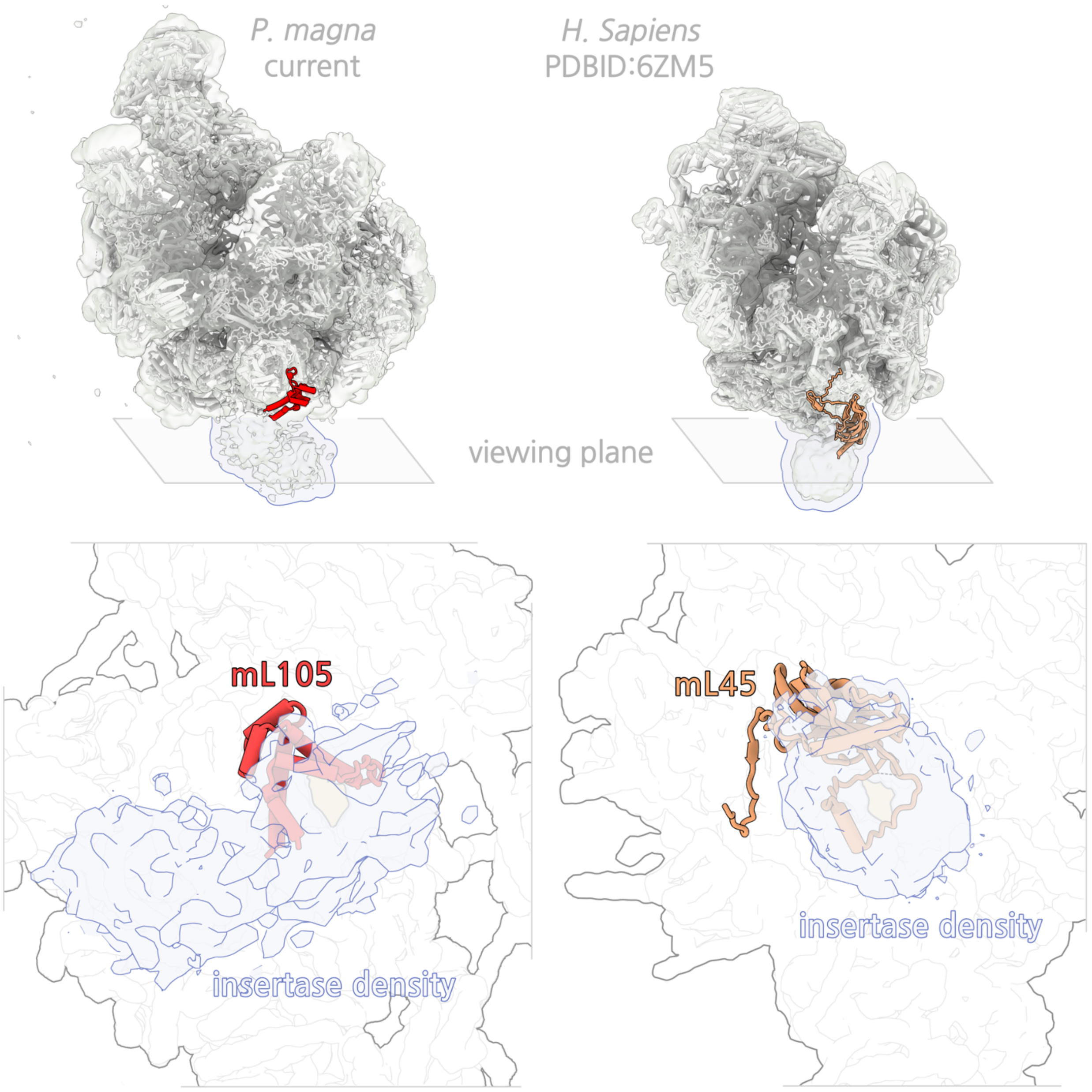

Supplementary Fig. 8: Positioning of insertase density.
View orthogonal to the membrane plane of the exit tunnel region in *P. magna* and *H. sapiens*. Protein in closest proximity to additional putative insertase density colored. Low resolution insertase density filtered to 10 Å and colored blue.

Supplementary Table 1: Cryo-EM data collection, refinement and validation statistics.

	Consensus (EMDB-15100) (PDB 8A22)						Classical tRNA (EMDB-15579) (PDB 8APN)			Hybrid tRNA (EMDB-15577) (PDB 8APO)		
**Data collection and processing**												
Magnification	130,000											
Voltage (kV)	300											
Electron exposure ($e^-/Å^2$)	30.0											
Defocus range (μm)	-0.2 to -2.0											
Pixel size (Å)	0.84											
Initial particle (no.)	2,999,000											
Symmetry imposed	C1											
Final particle (no.)	184,300						76,000			59,000		
Resolution ranges (Å)												
FSC threshold 0.143												
Solvent Mask	LSU	SSU	HEAD	TAIL	EXT	BP	LSU	SSU	HEAD	LSU	SSU	HEAD
Highest	2.74	2.82	2.80	3.05	3.72	3.15	3.02	3.10	3.11	2.93	3.00	2.96
Median	3.10	3.06	3.16	3.33	4.88	3.72	3.54	3.48	3.73	3.36	3.27	3.38
Lowest	14	9	9	8	15	9	15	14	11	15	11	11
Map sharpening B-factor ($Å^2$)	-55.3	-53.5	-58.2	-67.9	-106.7	-78.4	-55.1	-58.0	-53.8	-54.2	-49.0	-60.7
**Refinement**												
Initial model used (PDB code)	6Z1P, 6XYW, 6YWX, 5MRC, 3J9M, 6HIV											
Model composition												
Non-hydrogen atoms	196,555						198,408			197,825		
Protein residues	17,557						17,556			17,547		
Nucleotides	2,685						2,773			2,748		
Ligands												
Mg / K / ATP / Zn	111 / 31 / 1 / 1 / 1						111 / 31 / 1 / 1 / 1			111 / 30 / 1 / 1 / 1		
B factors ($Å^2$)												
(min/max/mean)	9.06 / 95.59 / 41.45						9.06 / 95.59 / 41.45			7.62 / 95.59 / 48.84		
Protein	4.48 / 114.32 / 34.94						4.48 / 114.32 / 34.94			0.99 / 153.27 / 42.63		
Nucleotide	11.60 / 76.73 / 28.84						11.60 / 76.73 / 28.84			10.90 / 88.34 / 35.46		
Ligand												
R.m.s. deviations												
Bond lengths (Å)	0.004						0.003			0.003		
Bond angles (°)	0.793						0.750			0.752		
Validation												
MolProbity score	0.96						0.9			0.92		
Clashscore	1.93						1.55			1.7		
Poor rotamers (%)	0.45						0.4			0.41		
CaBLAM outliers (%)	0.94						0.97			0.94		
Ramachandran plot												
Favored (%)	98.48						98.76			98.83		
Allowed (%)	1.47						1.22			1.15		
Disallowed (%)	0.05						0.02			0.02		

Supplementary Table 2: Mitoribosomal proteins of *Polytomella*.

Chain ID	Nomenclature	Chain length	ORF length	Modeled residue ranges relative to predicted ORFs	Comments
Aa	uL2m	306	392	57-362	
Ab	uL3m	306	407	98-403	
Ac	uL4m	303	359	48-350	
Ad	uL5m	193	236	48-233	
Ae	uL6m	242	318	161-313	
Af	uL9m	56	281	91-146	
Ah	uL13m	186	207	17-181	
Ai	uL14m	121	123	3-123	
Aj	uL15m	206	283	74-280	
Ak	uL16m	166	268	102-267	
Al	bL17m	173	235	6-179	
Am	uL18m	114	115	2-115	
An	bL19m	170	225	53-222	
Ao	bL20m	117	132	7-112	
Ap	bL21m	200	200	18-189	
Aq	uL22m	188	256	68-255	
Ar	uL23m	155	214	52-191	
As	bL24m	115	182	22-73, 80-105, 145-182	
At	bL25m	253	289	36-218	
Au	bL27m	142	203	60-201	
Av	bL28m	129	177	43-171	Model missing N-terminal residues
Aw	uL29m	123	173	50-172	Model missing C-terminal residues
Ax	uL30m	176	180	4-179	
Ay	bL31m	72	109	29-101	
Az	bL32m	59	148	76-119	
AA	bL33m	50	71	21-70	
AB	bL34m	50	164	114-163	
AC	bL35m	139	205	64-204	
AD	bL36m	46	102	7-52	Zn binding
AE	mL40	92	163	87-162	
AF	mL41	93	104	8-101	
AG	mL43	121	112	8-128	
AH	mL46	176	138	2-138	
AI	mL63	64	90	9-72	
AJ	mL64	122	137	6-131	
AK	mL87	139	169	28-166	
AL	mL116	394	501	76-295	HEAT
AM	mL116	419	501	82-501	HEAT
AN	mL116	420	501	82-501	HEAT
AO	mL118	377	525	94-421, 423-454, 458-476	HEAT
Xa	mL120	199	256	54-252	HEAT
Xb	mL121	244	268	16-265	HEAT
Xc	mL122	57	97	37-93	
Xd	mL123	413	527	63-474	HEAT
Xe	mL124	483	564	70-503	HEAT
Xf	mL125	201	218	21-229	Missing C-terminal sequence
Xg	mL126	410	518	85-198, 206-226, 254-410	HEAT, missing C-terminal sequence
Xh	mL127	143	352	57-97, 210-352	
Xi	mL128	24	32	9-32	
Xj	mL129	71	78	7-78	
Ba	bS1m	242	302	57-299	
Bb	uS2m	236	292	24-138, 169-290	
Bc	uS3m	289	417	142-348, 367-387	
Bd	uS4m	221	272	51-272	
Be	uS5m	228	376	145-373	

Bf	uS6m	119	123	5-123	
Bg	uS7m	112	220	103-115	
Bh	uS8m	374	442	5-202, 233-409	
Bi	uS9m	282	376	243-376	
Bj	uS10m	401	553	111-301, 316-400, 429-553	Missing C-terminal sequence
Bk	uS11m	116	204	98-204	
Bl	uS12m	123	239	116-239	
Bm	uS13m	113	127	6-118	
Bn	uS14m	118	128	11-128	
Bo	uS15m	167	184	62-229	Missing C-terminal sequence
Bp	bS16m	123	135	13-135	
Bq	uS17m	130	160	15-116	
Br	bS18m	90	316	205-295	
Bs	bS19m	92	120	6-98	
Bt	bS21m	75	91	17-91	
Bu	mS23	167	182	6-173	
Bv	mS26	164	229	66-229	
Bw	mS29	349	448	99-448	
Bx	mS31	621	658	1-112, 223-419, 425-645	
By	mS33	80	133	44-211	
Bz	mS34	119	77	4-81	
BA	mS35	176	207	31-207	
BB	mS37	84	89	11-71, 77-84	
BC	mS38	31	148	118-148	
BD	mS45	270	137	45-123	
BE	mS106	171	249	115-282	HEAT
BF	mS107	370	441	56-183, 200-441	HEAT
Ya	uS4m-2	180	241	62-241	Fragment of S4
Yb	mS108	50	112	36-86	
Yc	mS109	159	258	103-262	
Yd	mS110	95	146	52-146	
Ye	uS3m-2	106	127	4-58, 74-124	
Yf	mS111	150	239	63-213	
Yg	mS112	67	71	4-70	
Yh	mS113	65	104	17-81	
Yi	mS114	132	137	6-138	
Yj	mS115	386	849	289-654, 665-679	Unidintified ligand
Yk	mS116	92	148	8-64, 96-107	
Yl	uS7m-2	84	121	31-49, 56-140	Fragment of S7
Ua	UNK1	32			Helical pair in LSU, possible extension from existing protein
Ub	mL105	131			
Ud	UNK4	43			Final helix of helical back protuberance bundle
Ue	UNK5	47			Chain in interface between body platform and head, likely merged with exisiting proteins
Uf	UNK6	73			Helical fold on head extension possibly merged with with mS31 and/or UNK8
Ug	UNK7	63			Chain in interface between body platform and head, likely merged with exisiting proteins
Uh	UNK8	48			Helical fold on head extension possibly merged with with mS31 and/or UNK6
Ui	UNK9	48			Small peptide on SSU platform, likely merged with UNK10
Uj	UNK10	9			Small peptide on SSU platform, likely merged with UNK9
Uk	UNK11	23			Chain in interface between body platform and head, likely merged with exisiting proteins
Ul	UNK12	16			Chain in interface between body platform and head, likely merged with exisiting proteins
Um	UNK13	11			Chain in interface between body platform and head, likely merged with exisiting proteins
